# Therapeutic benefit of Muse cells in a mouse model of amyotrophic lateral sclerosis

**DOI:** 10.1038/s41598-020-74216-4

**Published:** 2020-10-13

**Authors:** Toru Yamashita, Yoshihiro Kushida, Shohei Wakao, Koh Tadokoro, Emi Nomura, Yoshio Omote, Mami Takemoto, Nozomi Hishikawa, Yasuyuki Ohta, Mari Dezawa, Koji Abe

**Affiliations:** 1 Department of Neurology, Okayama University Graduate School of Medicine, Dentistry and Pharmaceutical Sciences, Okayama, Japan; 2 Department of Stem Cell Biology and Histology, Tohoku University Graduate School of Medicine, Sendai, Japan

**Keywords:** Mesenchymal stem cells, Neurological disorders

## Abstract

Amyotrophic lateral sclerosis (ALS) is a fatal neurodegenerative disease characterized by progressive motor neuron loss. Muse cells are endogenous reparative pluripotent-like stem cells distributed in various tissues. They can selectively home to damaged sites after intravenous injection by sensing sphingosine-1-phosphate produced by damaged cells, then exert pleiotropic effects, including tissue protection and spontaneous differentiation into tissue-constituent cells. In G93A-transgenic ALS mice, intravenous injection of 5.0 × 10^4^ cells revealed successful homing of human-Muse cells to the lumbar spinal cords, mainly at the pia-mater and underneath white matter, and exhibited glia-like morphology and GFAP expression. In contrast, such homing or differentiation were not recognized in human mesenchymal stem cells but were instead distributed mainly in the lung. Relative to the vehicle groups, the Muse group significantly improved scores in the rotarod, hanging-wire and muscle strength of lower limbs, recovered the number of motor neurons, and alleviated denervation and myofiber atrophy in lower limb muscles. These results suggest that Muse cells homed in a lesion site-dependent manner and protected the spinal cord against motor neuron death. Muse cells might also be a promising cell source for the treatment of ALS patients.

## Introduction

Amyotrophic lateral sclerosis (ALS) is a devastating neurodegenerative disease characterized by progressive motor neuron loss. About 10% of ALS patients have a genetically inherited form associated with mutations in Cu/Zn superoxide dismutase (SOD1)^1–3^, TAR DNA binding protein 43 (TDP-43)^4,5^, and a hexanucleotide repeat expansion of the C9ORF72 gene^6,7^. In addition to an oral drug riluzole, a free radical scavenger edaravone was recently approved as a new anti-ALS drug^8,9^. However, the therapeutic benefits of those treatments are still greatly limited, which demands a novel therapeutic strategy for ALS.

Multilineage-differentiating stress-enduring (Muse) cells are endogenous pluripotent-like stem cells collectable as cells positive for the pluripotent stem cell surface marker, stage-specific embryonic antigen (SSEA)-3. They are normally located in the bone marrow, peripheral blood, and connective tissues of organs and are thus non-tumorigenic^10–13^. Muse cells are unique for several reasons: they recognize damaged tissue and selectively accumulate at the site of damage by intravenous injection because they express sphingosine-1-phosphate (S1P) receptor 2, which recognizes the S1P produced by damaged/apoptotic cells; after homing to the damaged site, Muse cells replace damaged/apoptotic cells by spontaneous differentiation into the damaged/apoptotic cell-type, and contribute to tissue repair, as shown by animal models of stroke, acute myocardial infarction, epidermolysis bullosa, chronic kidney disease and liver cirrhosis^14–18^. Besides their effects on tissue repair, Muse cells have pleiotropic effects including neovascularization, immunomodulation, trophic-, anti-apoptotic-, and anti-fibrotic effects^18,19^. Another important and unique feature is that allogeneic-Muse cells escape host immunorejection after intravenous administration and survive in the host tissue as differentiated cells for over 6 months, even without immunosuppressive treatment^18^. This is partly explained by the expression of human leukocyte antigen (HLA)-G, a histocompatibility antigen that mediates immune tolerance in the placenta^18^. Based on these properties, intravenously administered allogenic-Muse cells have been applied to clinical trials for acute myocardial infarction, stroke, spinal cord injury, epidermolysis bullosa and neonatal cerebral palsy after approval of the relevant regulatory authority, all without HLA matching or long-term immunosuppressant treatment^20^. Since Muse cells are able to target damaged tissues, the number of cells required for treatment is at an order of magnitude less than that in mesenchymal stem cells (MSCs)^21^. For these reasons, we examined a possible therapeutic potential of Muse cells for the ALS animal model.

## Results

**Figure 1:**
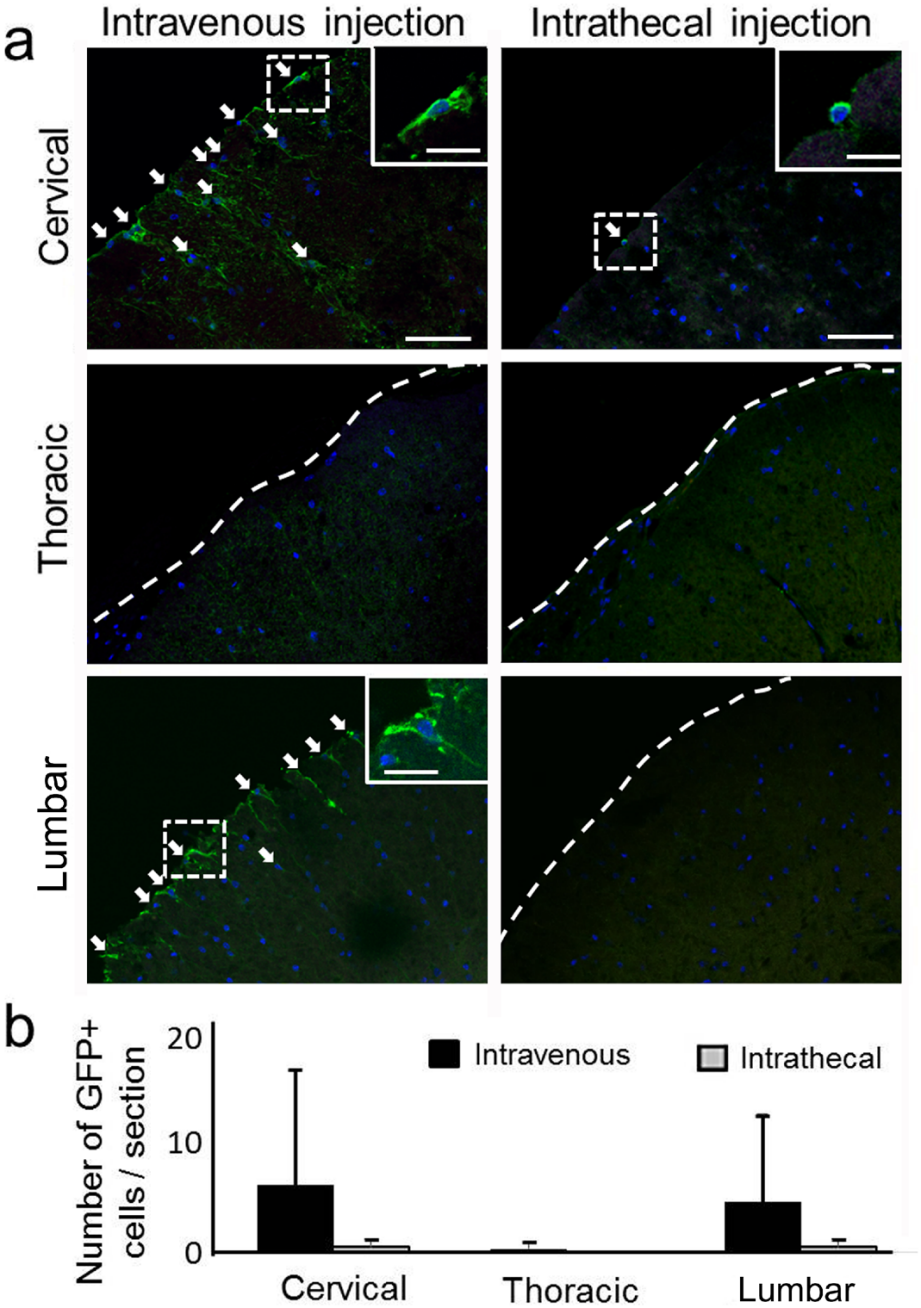
(**a**) Distribution of GFP-labeled Muse cells in the spinal cord at 7 days after intravenous (IV) or intrathecal (IT) injection. The dotted lined boxes in panels indicate the zone of higher magnification in each panel. Of note, only IV injection delivered many GFP-labeled Muse cells to the pia-mater and underneath white matter of both cervical and lumbar spinal cords. Scale bars: 100 μm (**a**); 20 μm (**a**, bracket). (**b**) Number of GFP-labeled Muse cells were higher after IV injection than after IT injection.

To determine the route of administration, homing of GFP-Muse cells after IV- and IT-injections was compared by histological analysis of the spinal cord of G93A mice at 7 days after injection. One mouse died a day after IT injection, probably due to the high invasiveness of this method. The pilot study demonstrated that the number of GFP-Muse cells was consistently low or neglectable in the cervical, thoracic and lumbar spinal cord in the IT-injection group, but was significantly higher in the cervical and lumbar spinal cord of the IV-injection group. Moreover, those GFP-Muse cells were mainly located at the pia-mater and underneath white matter. GFP-Muse cells were rarely detected in the thoracic spinal cord, even after IV-injection (Table 1, Fig. 1a,b). Consequently, IV-injection was selected as the route of administration in the following experiments.

**Table 1:** The number of GFP-labeled Muse cells detected in spinal cords (in vivo comparative experiment between IV and IT).

	IV (n = 3)			IT (n = 2)		
	Animal no	Pia mater-white matter	Ventral horn	Animal no	Pia mater-white matter	Ventral horn
Cervical	IV-➀	+~++	−	IT-➀	−~+	−
	IV-➁	+~ +++	+~++	IT-➁	−~+	−
	IV-➂	−	−			
Thoracic	IV-➀	−	−	IT-➀	−	−
	IV-➁	−	−	IT-➁	−~±	−
	IV-➂	−~±	−			
Lumber	IV-➀	+~+++	−	IT-➀	−	−
	IV-➁	−	−	IT-➁	−	−
	IV-➂	−	−			

−, no GFP-positive cells; +, 1–4 per section; ++, 5–9 per section; +++, > 10 per section.

**Figure 2:**
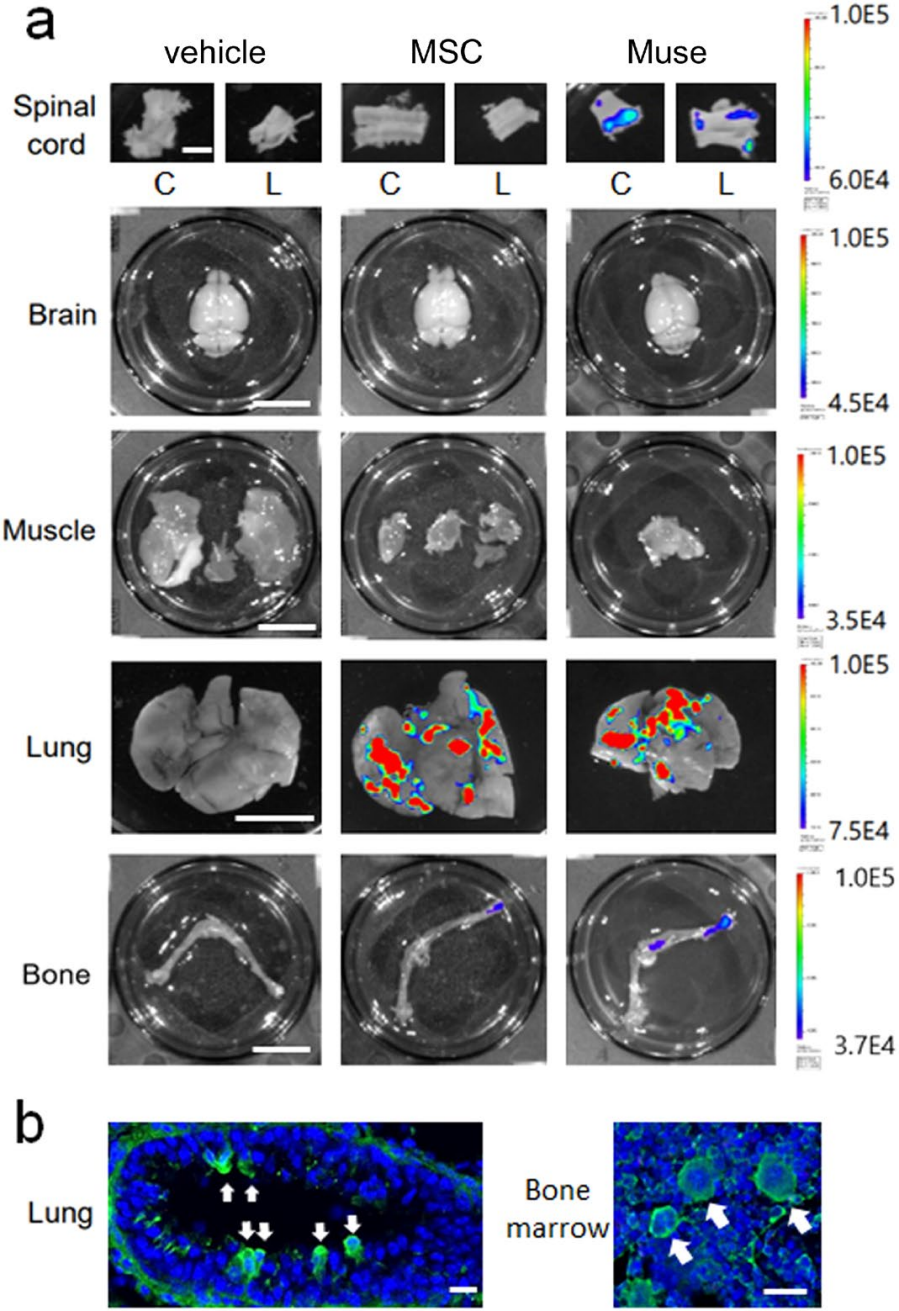
(**a**) Distribution of Nano-lantern-labeled human MSCs and Muse cells in the spinal cord, brain, muscle, lung, and leg bone 7 days after IV administration. Of note, only Muse cells were detected in the spinal cord (c; cervical spinal cord, l; lumbar spinal cord) (**b**) Nano-lantern-labeled Muse cells were found in the pulmonary vessel lumen (left panel, arrows), and in the bone marrow (right panel, arrows). Scale bar in (**a**, spinal cord) 2 mm, in (**a**, others) 1 cm, and in (**b**) 20 μm.

In order to examine the in vivo dynamics of MSCs and Muse cells after IV-injection, Nano-lantern-labeled cells were used. In the MSC group, an intense signal was detected in the lung and a trace signal in the femur bone while not in other organs including the brain, cervical and lumbar spinal cord at day 7. In the Muse group, the signal was detected in the cervical and lumbar spinal cord (Fig. 2a, top right) as well as in the lung, while not in the brain (Fig. 2a, middle right). The signal in the femur bone was higher in the Muse group than in the MSC group. Histological analysis confirmed the presence of Nano-lantern-Muse cells in the pulmonary vessel lumen and the bone marrow (Fig. 2b). The signal was consistently under the detection limit in all organs inspected in the vehicle group (Fig. 2a).

**Figure 3:**
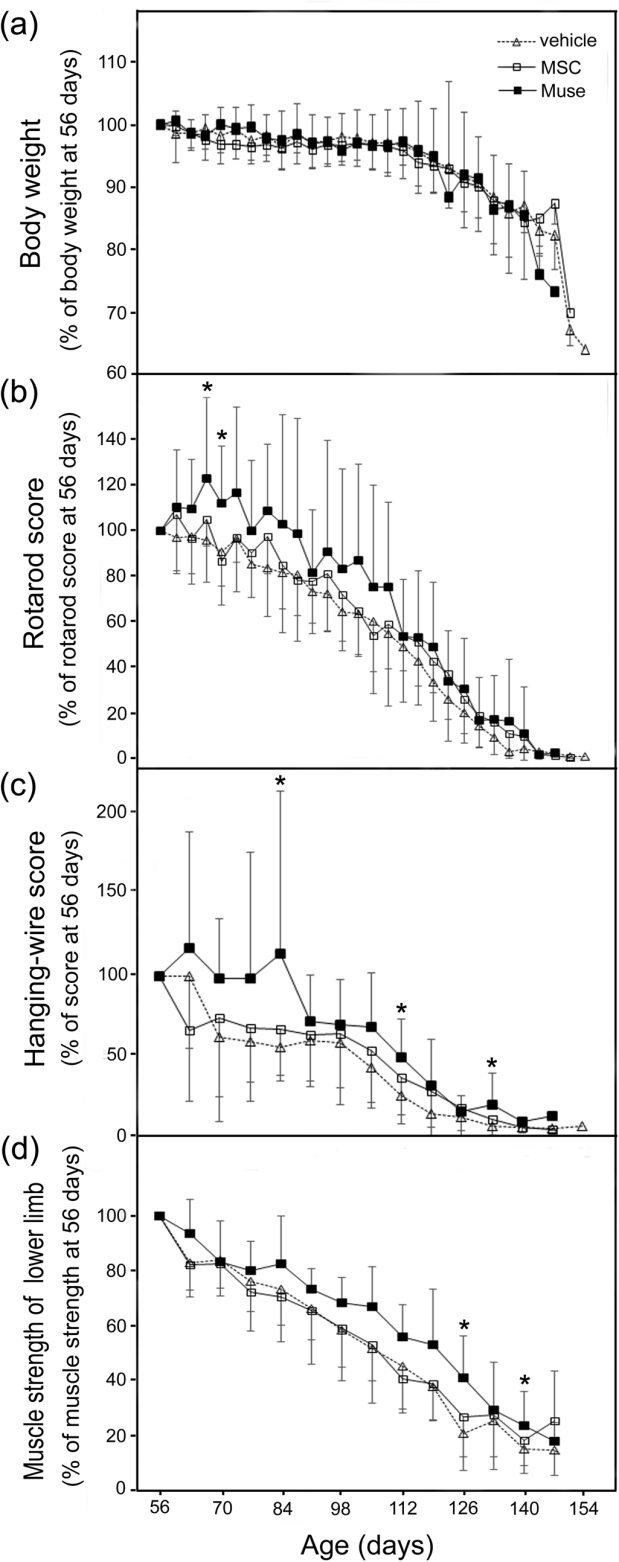
Clinical analysis of G93A mice treated with vehicle (n = 10), MSCs (n = 9), and Muse cells (n = 9): (**a**) body weight, (**b**) rotarod test, (**c**) hanging-wire test, and (**d**) muscle strength of lower limbs. Compared with the vehicle, IV treatment of Muse cells showed significant improvement in the rotarod and hanging-wire scores, and muscle strength of lower limbs (*p < 0.05, vs vehicle).

The mean survival time showed no significant difference among the three groups (vehicle; 144.4 ± 8.0 days, MSC; 143.9 ± 6.9 days, Muse cells; 142.6 ± 6.7 days). There was also no statistical difference in body weight among the three groups throughout the entire period (Fig. 3a). In contrast, the rotarod test showed an alleviation in the Muse group at both 67 (*p* = 0.036) and 70 days (*p* = 0.049), significantly higher from the vehicle groups (Fig. 3b). In addition, both the hanging-wire score at 84, 112, and 133 days and muscle strength of the lower limbs at 126 and 140 days also improved significantly, but only in the Muse group compared to the vehicle group (Fig. 3c,d).

**Figure 4:**
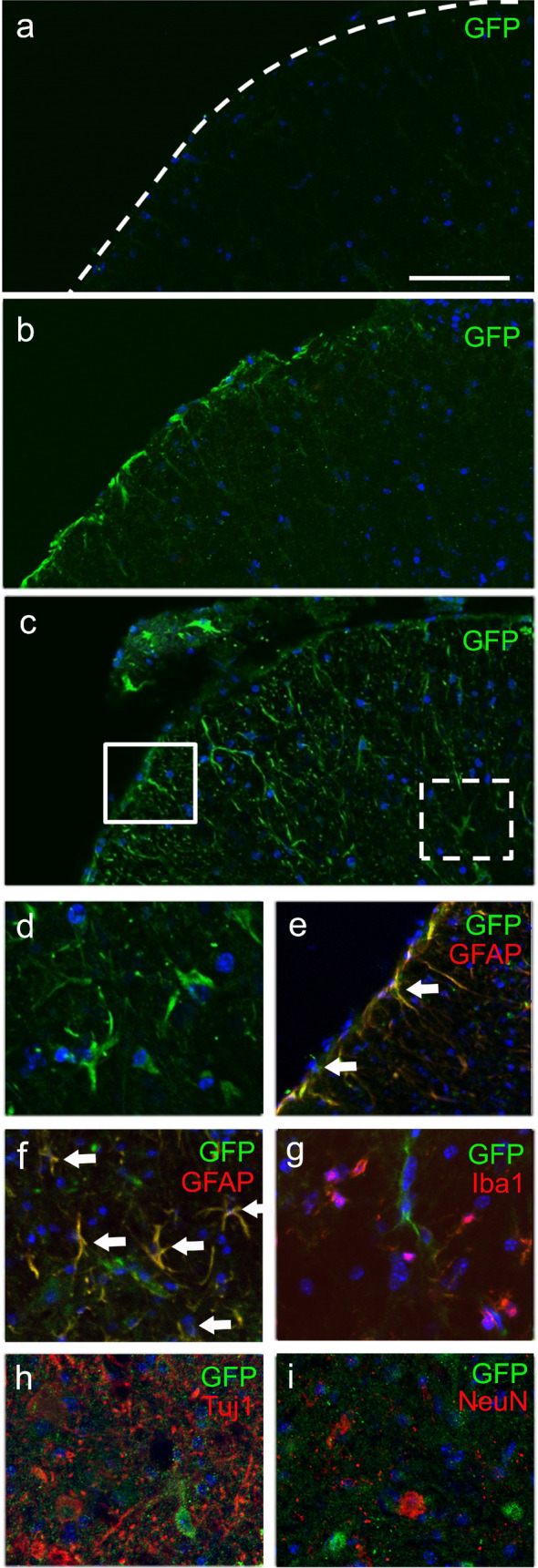
(**a**) No GFP-positive cells in the lumbar spinal cord of G93A mice treated with vehicle. (**b**) Several GFP-positive cells were found only in the pia-mater in the MSC group, and (**c**) many more GFP-positive cells detected from the pia-mater to the ventral horn in the Muse cells group. The boxed areas in (**c**) are magnified in (**e**) (solid box) and (**f**) (dotted box), respectively. Muse cells treatment showed evident GFP-positive cells (**d**) and GFP/GFAP double-positive cells in the pia-mater (**e**, arrows) and ventral horn (**f**, arrows) exhibiting morphology typical of astroglia (arrows), but no double-positive cells for GFP plus microglial marker Iba-1 (**g**), or neuronal markers such as Tuj1 (**h**) and NeuN (**i**). Scale bars: 100 μm (**a**); 50 μm (**d**).

In the lumbar spinal cord, GFP-positive cells were undetectable in the vehicle group (Fig. 4a), whereas a small number of GFP-positive cells were observed in the MSC group at the end-stage (22 weeks-old mice) (Fig. 4b). In the Muse group, GFP-positive cells were recognized at the spinal pia-mater (Fig. 4c, solid square), underneath white matter, and at the ventral horn (Fig. 4c, dotted square, 4d). In addition, 85.7% (180 out of 210 GFP-positive cells) of those cells co-expressed the astrocytic marker GFAP located at both the pia-mater (Fig. 4e) and ventral horn (Fig. 4f). The remainder of GFP-positive cells (14.3%) did not stain positively for microglial marker Iba-1 (Fig. 4g, 0/120 GFP-positive cells), or neuronal markers Tuj1 (Fig. 4h, 0/97 GFP-positive cells) and NeuN (Fig. 4i, 0/109 GFP-positive cells), suggesting that the majority of IV-injected Muse cells spontaneously differentiated mainly into astrocyte-lineage cells after homing into the lumbar spinal cord.

**Figure 5:**
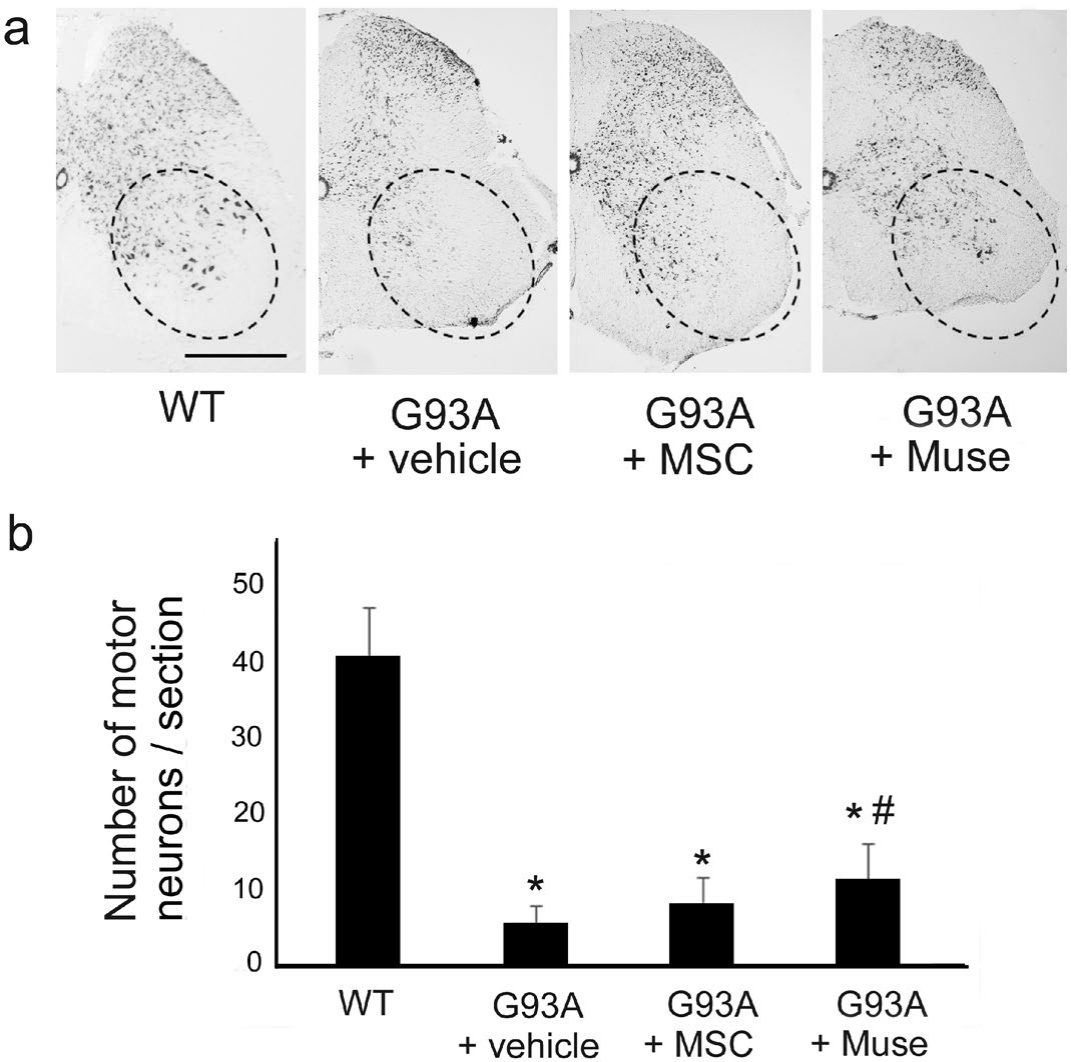
Number of Nissl-stained motor neurons in the lumber spinal cord showed a significant decrease in G93A mice (*p < 0.05, vs wild type = WT), but a significant improvement following IV treatment of Muse cells (^#^*p* < 0.05, vs vehicle). Scale bar: 500 μm (**a**).

When compared with the wild type (WT) group, the number of surviving motor neurons of the ventral horn was significantly lower in the vehicle, MSC and Muse groups at the end-stage (22 weeks-old mice) (WT, Fig. 5a,b, *p < 0.05). However, the Muse group displayed significantly more motor neurons than the vehicle group (Fig. 5a,b, ^#^*p* < 0.05). There were no statistically significant differences between the vehicle and MSC groups (Fig. 5a,b).

**Figure 6:**
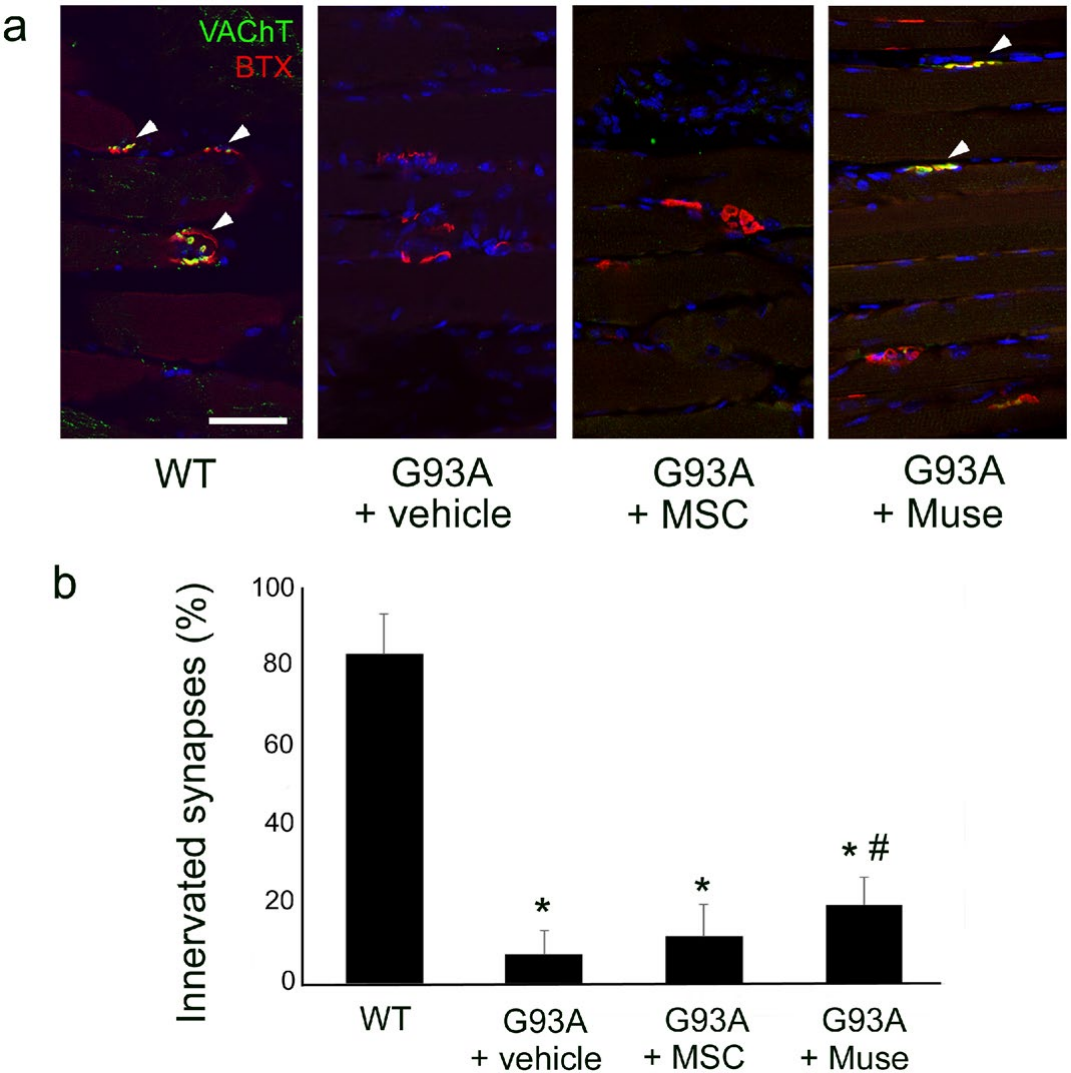
Staining of the neuromuscular junction (NMJ) showing denervation in the tibialis anterior muscle of G93A mice, and recovery in the Muse cells group (VAChT-positive motor terminals; green, acetylcholine receptors stained with BTX; red, arrowheads, *p < 0.05, vs WT, ^#^*p* < 0.05, vs vehicle). Scale bar: 50 μm (**a**).

**Figure 7:**
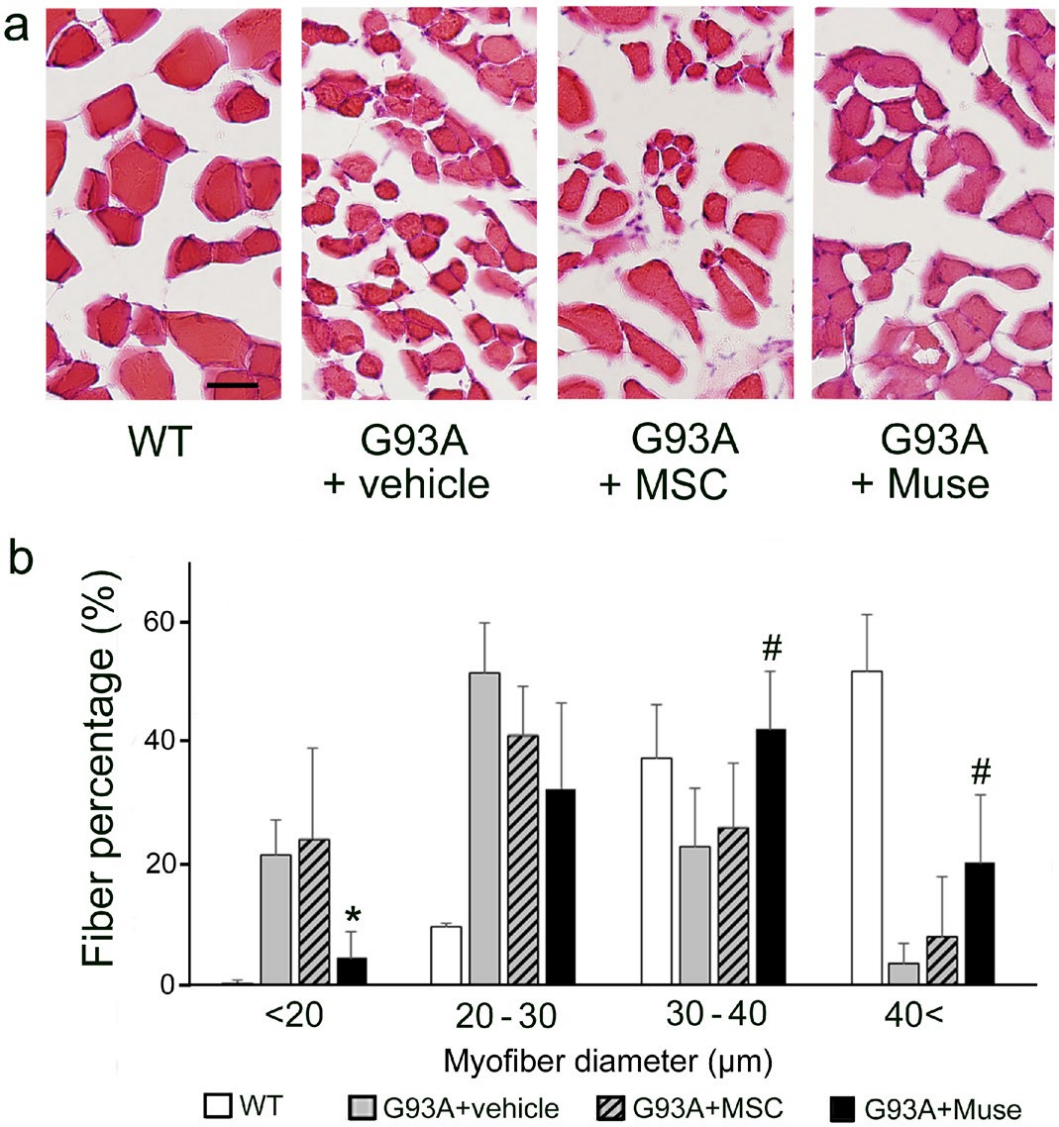
HE-stained neurogenic myofiber atrophy in the tibialis anterior muscle of G93A mice, and a significant improvement following treatment with Muse cells (*p < 0.05, vs vehicle, ^#^*p* < 0.05, vs MSC). Scale bar: 50 μm (**a**).

In the tibialis anterior muscle, the number of innervated synapses was significantly lower in the vehicle, MSC and Muse groups than in the WT (Fig. 6a,b, *p < 0.05). However, values in the Muse group recovered to levels comparable with the vehicle group (Fig. 6a,b, ^#^*p* < 0.05). An analysis of myofiber size demonstrated severe neurogenic myofiber atrophy in the vehicle and MSC groups, which improved in the Muse group, with statistically comparable values with both the vehicle (*p < 0.05) and MSC (^#^*p* < 0.05) groups (Fig. 7a,b).

## Discussion

The present study is the first report to demonstrate that multiple IV administration of Muse cells improved clinical scores in the rotarod, hanging-wire and muscle strength of lower limbs in the ALS model G93A Tg mice. IV-injected Muse cells homed to the lumbar spinal cord, lung and bone (Fig. 2a,b). In the lumbar spinal cord, GFP-positive Muse cells predominantly expressed astroglial marker GFAP and exhibited a glia-like morphology at the end-stage (22 weeks-old, Fig. 4c–f). In addition, the number of surviving motor neurons in the lumber spinal cord was significantly higher than the vehicle group (Fig. 5a,b), which might have led to the alleviation of both the denervation and myofiber atrophy in the lower limb muscles (Figs. 6, 7).

Previous studies reported that IT-injected MSCs had the potential to prolong the life span and delay the decline of motor performance in the ALS animal model^22,23^. On the other hand, the present study data showed that IV-injection was superior to IT-injection in terms of delivering Muse cells to the critical therapeutic target, namely the spinal cord, in ALS model mice (Table 1, Fig. 1a,b). It was also reported that IV-injected Muse cells clearly showed a therapeutic effect in functional recovery of the acute stroke mice model, in a dose-dependent manner^24^. IV-administration has greater advantages over IT-administration because of easy accessibility, less-invasiveness and less-burden for patients. These may allow repeated administration of Muse cells to ALS patients. Recent studies demonstrated that Muse cells that express sphingosine-1-phosphate receptor 2 (S1PR2) can specifically home to a damaged site by sensing sphingosine-1-phosphate (S1P) produced by damaged cells^18^. S1P is a general damage signal common to all organs because it is produced by the phosphorylation of sphingosine, one of the components of the cell membrane. Therefore, Muse cells could home to the spinal cord of ALS mice by IV-injection.

Nano-lantern imaging demonstrated a stronger signal of Muse cells in the femur bone than MSCs (Fig. 2a). Bone marrow abnormality was reported in ALS^25^. Therefore, Muse cells selectively and actively accumulated to the femur bone marrow as well as to the spinal cord in a lesion-dependent manner, in contrast to passive entrapment in lung capillaries. Another interesting point is that IV-injected Muse cells migrated to the spinal pia-mater, underneath white matter, and to the ventral horn (Fig. 4c–f), suggesting homing of Muse cells into the spinal cord via pial perforating arteries. On the other hand, IT-injected Muse cells were scarcely detected in the spinal cord (Fig. 1a,b). These results suggest that homing factors for Muse cells were not mainly paracrined to the spinal subarachnoid space, but were endocrined to the circulating system^25–28^.

In animal disease models, IV-injected Muse cells spontaneously differentiated into tissue-constituent cells after specific homing to damaged tissues, i.e., they differentiated into neuronal cells and oligodendrocytes in a mouse stroke model^16^. In contrast to stroke, ALS is chronic and progressive and the state of the disease is completely different to that of a stroke. IV-injected Muse cells did not differentiate into a neuronal-lineage but mainly into an astroglial-lineage in the spinal cord of ALS mice. Reactive astrocytes are an important therapeutic target of ALS^29^, and the microenvironmental signal of the ALS mice spinal cord may differentiate Muse cells into GFAP-positive astrocytes, especially into A2 astrocytes, which might secrete molecules that provide neurotrophic support and modulate inflammatory responses^30^. Muse cells themselves can produce various neurotrophic factors, including brain-derived neurotrophic factor (BDNF), hepatocyte growth factor (HGF), vascular endothelial growth factor (VEGF), insulin-like growth factor 1 (IGF-1), epidermal growth factor (EGF), prostaglandin E2 (PGE2), and angiopietin-1 (Ang1)^16–19,31^. Therefore, they might have supplied beneficial factors to motor neurons and astrocytes, preventing myofiber atrophy in the ALS model. In addition, clinical trials for acute myocardial infarction, stroke, spinal cord injury, epidermolysis bullosa and neonatal cerebral palsy are currently being conducted, all via an IV-drip of donor Muse cells without HLA-matching or long-term immunosuppressive drugs. In a clinical trial of acute myocardial infarction, safety and remarkable cardiac function recovery were reported^21^.

Overall, the present study successfully achieved, for the first time, the systemic administration of Muse cells that showed a significant clinical benefit for the ALS mice model, in which IV-injected Muse cells preferentially migrated to the spinal cord, supplied astroglia, supported motor neuron survival and suppressed myofiber atrophy. Muse cells can be a promising cell resource for the treatment of ALS patients.

## Materials and methods

### Animals and experimental groups

The data that support the findings of this study are available from the corresponding author upon reasonable request. All animal experiments were approved by the Institutional Animal Care and Use Committee of Okayama University (OKU-2019289), and performed in accordance with the guidelines of Okayama University on animal experiments. Transgenic (Tg) mice with the G93A human SOD1 mutation (G1H/ +) were obtained from Jackson Laboratories (Bar Harbor, ME, USA)^2^ and maintained as hemizygotes by mating Tg males with C57BL/6J females. A comparative experiment between intravenous (IV) and intrathecal (IT) injections used three animals for each group, Nano-lantern ex-vivo imaging used two animals and for the vehicle, MSC and Muse cells, respectively. To evaluate therapeutic efficacy by IV-injection, the vehicle group used 10 mice (five males and five females), MSC nine mice (four males and five females), and Muse cells nine mice (five males and four females).

### Preparation of GFP-labeled MSC and Muse cells

GFP-labeled MSCs were prepared by labelling human bone marrow-MSCs (Lonza, Basel, Switzerland) with lentivirus GFP as previously described^32^. GFP-labeled Muse cells were isolated as SSEA-3-positive cells from GFP-MSCs by fluorescence-activated cell sorting (FACS) as previously described^10,11^. We confirmed the viability of Muse cells and MSCs by trypan blue or PI staining every time, and found that ~ 98% of the cells were alive. The above GFP-MSCs and -Muse cells were frozen in a Beissel freezing container (Nihon Freezer, Tokyo, Japan), and kept in liquid nitrogen until use.

### In vivo comparative cell transplantation for injection route

The above GFP-labeled Muse cells (2.0 × 10^5^) in 250 μl of Hank's balanced salt solution (HBSS, pH 7.4) were injected into the tail vein for IV or into cisterna magna for intrathecal injection (IT) as previously described^33,34^. At 7 days after injection, all animals were sacrificed.

### Ex-vivo dynamics with nano-lantern

Nano-lantern-labeling with bioluminescence resonance energy transfer (BRET) efficacy is a bright luminescent protein that allows the detection of even a small number of transplanted cells^35^. Human bone marrow-MSCs (Lonza) were labeled with Nano-lantern as previously described^35^. Nano-lantern-labeled Muse cells were isolated from Nano-lantern-labeled MSCs as SSEA-3-positive cells by FACS. For ex-vivo dynamics of IV-injection, vehicle (HBSS), Nano-lantern-labeled-MSCs and -Muse cells (1.0 × 10^5^ cells/250 μl) were intravenously injected when mice were 14 weeks old. After 7 days, animals were sacrificed under deep anesthesia and analyzed as described previously^24^.

### Evaluation of therapeutic efficacy

Vehicle (HBSS), GFP-MSCs and GFP-Muse cells (both 5.0 × 10^4^ cells/250 μl) were injected into the tail vein of each animal within one min. Because our and other groups confirmed that G93A Tg mice were reported to exhibit early onset at around 56 days of age^36^, we decided to start cell administration at 56 days of age, and continue the administration once a week until 119 days (total of 10 injections) with the same number of cells. An immunosuppressive compound, cyclosporine A (10 mg/kg/day, Novartis International, Basel, Switzerland), was also applied intraperitoneally (IP) to all animals immediately after administration in the vehicle, MSC, or Muse groups every other day until sacrifice. Survival was checked every day, and body weight (BW) and the rotarod score were measured twice a week from 56 days of age. The rotarod test and wheel-running activity were performed according to our previous methods^34,37^. The hanging-wire test was evaluated once a week as a measure of muscular strength and coordination as previously reported^38^. Muscle strength of lower limbs was measured once a week by using a precision spring scale (Ooba Keiki Co., Tokyo, Japan).

### Histological analysis

The time point at which mice were unable to roll over within 15 s of being pushed onto their side was recorded as the time of death for sacrifice, at the end-stage (22 weeks-old mice). Each animal was deeply anesthetized by intraperitoneal injection of pentobarbital (20 mg/kg), and then subjected to sampling as described before^24^. Primary antibodies used were: goat anti-GFP antibody (1:500, Abcam, Cambridge, UK); rabbit anti-GFP antibody (1:500, MBL, Woburn, USA); rabbit anti-Iba1 antibody (1:500, Wako, Osaka, Japan); mouse anti-betaIII tubulin (Tuj1) antibody (1:100, Santa Cruz Biotechnology); rabbit anti-glial fibrillary acidic protein (GFAP) antibody (1:500, Dako, Glostrup, Denmark); mouse anti-NeuN antibody (1:100, Millipore, MA, USA); goat anti-vesicular acetylcholine transporter (VAChT) antibody (1:200, Thermo Scientific). Secondary antibodies used were either anti-goat, -rabbit or -mouse IgG conjugated with Alexa 488 or 546 (1:500, Alexa Fluor, Invitrogen, Carlsbad, CA, USA). Alpha-Bungarotoxin conjugated with Alexa 594 (1:500, Millipore) was also used to detect acetylcholine receptors.

For cell-type-marker/GFP double-labeling, 5–6 areas from the pia-mater to the anterior horn were randomly selected and analyzed. Nissl-stained motor neurons in L4–5 were counted using five transverse sections from each lumbar cord^39^. Cells larger than 20 μm with clear nucleoli in both ventral horns below a lateral line across the spinal cord from the central canal were counted as motor neurons^40^. For denervation, ~ 100 neuromuscular junctions (NMJ) from each mouse were analyzed. For myofiber size, ~ 180 myofibers from three hematoxylin and eosin (HE)-stained tibialis anterior muscle sections per mouse were analyzed by an investigator blinded to the treatment conditions.

### Statistical analyses

The investigators were blinded to the experimental group during data collection and analysis. The data that support the findings of this study are available from the corresponding author upon reasonable request. Data were analyzed in SPSS version 22.0.0.0 (IBM Corp., Armonk, New York, USA) and expressed as means ± SD. Therapeutic efficacy was evaluated by non-repeated measures analysis of variance (ANOVA) and Dunnett’s test. Histological data were analyzed by the Kruskall–Wallis test, followed by the Mann–Whitney U-test with a Bonferroni correction. In all statistical analyses, significance was assumed at *p* < 0.05.

## Abbreviations

Ang1: Angiopietin-1
ANOVA: Analysis of variance
BDNF: Brain-derived neurotrophic factor
BW: Body weight
BRET: Bioluminescence resonance
EGF: Epidermal growth factor energy transfer
FACS: Fluorescence-activated cell sorting
GFAP: Glial fibrillary acidic protein
GFP: Green fluorescence protein
HBSS: Hank’s balanced salt solution
HE: Hematoxylin and eosin
HGF: Hepatocyte growth factor
IGF-1: Insulin-like growth factor 1
IT: Intrathecal
IV: Intravenous
MSC: Mesenchymal stem cell
Muse: Multilineage-differentiating stress-enduring
NMJ: Neuromuscular junctions
PGE2: Prostaglandin E2
SOD1: Superoxide dismutase
S1P: Sphingosine-1-phosphate
S1PR2: Sphingosine-1-phosphate receptor 2
TDP-43: TAR DNA binding protein 43
Tg: Transgenic
VAChT: Vesicular acetylcholine transporter
VEGF: Vascular endothelial growth factor
WT: Wild type
