# Author Correction: Therapeutic benefit of Muse cells in a mouse model of amyotrophic lateral sclerosis

**DOI:** 10.1038/s41598-021-91963-0

**Published:** 2021-06-14

**Authors:** Toru Yamashita, Yoshihiro Kushida, Shohei Wakao, Koh Tadokoro, Emi Nomura, Yoshio Omote, Mami Takemoto, Nozomi Hishikawa, Yasuyuki Ohta, Mari Dezawa, Koji Abe

**Affiliations:** 1 Department of Neurology, Okayama University Graduate School of Medicine, Dentistry and Pharmaceutical Sciences, Okayama, Japan; 2 Department of Stem Cell Biology and Histology, Tohoku University Graduate School of Medicine, Sendai, Japan

Correction to: *Scientific Reports* 10.1038/s41598-020-74216-4, published online 13 October 2020

The original version of this Article contained an error in Figure 2, where the labels for the experimental group data were missing from panel 2a. The original Figure 2 and accompanying legend appear below.

**Figure 2:**
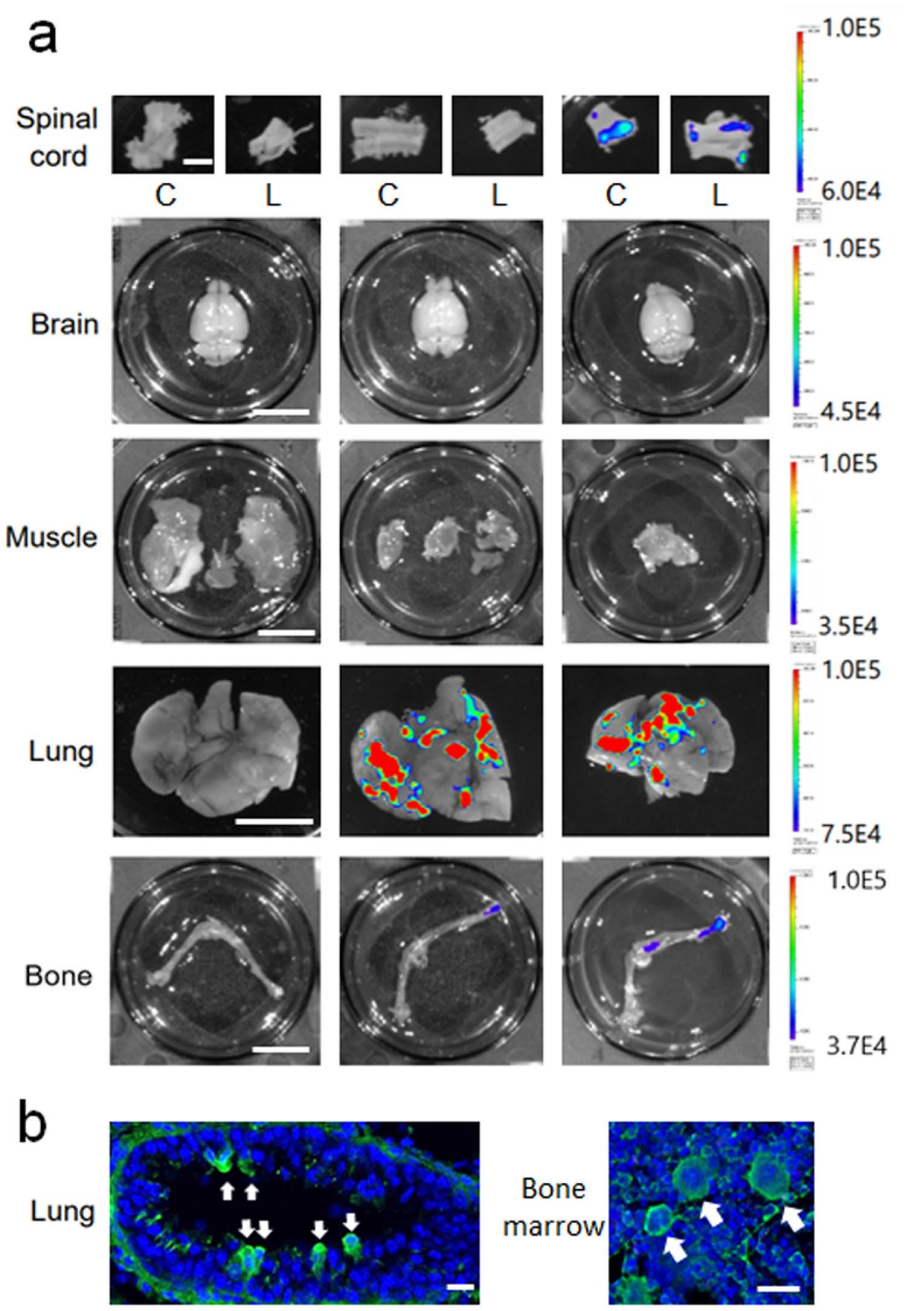
(**a**) Distribution of Nano-lantern-labeled human MSCs and Muse cells in the spinal cord, brain, muscle, lung, and leg bone 7 days after IV administration. Of note, only Muse cells were detected in the spinal cord (c; cervical spinal cord, l; lumbar spinal cord) (**b**) Nano-lantern-labeled Muse cells were found in the pulmonary vessel lumen (left panel, arrows), and in the bone marrow (right panel, arrows). Scale bar in (**a**, spinal cord) 2 mm, in (**a**, others) 1 cm, and in (**b**) 20 μm.

The original Article has been corrected.

